# Relationship of trainee dentists’ self-reported empathy and communication behaviors with simulated patients’ assessment in medical interviews

**DOI:** 10.1371/journal.pone.0203970

**Published:** 2018-12-20

**Authors:** Sho Watanabe, Toshiko Yoshida, Takayuki Kono, Hiroaki Taketa, Noriko Shiotsu, Hajime Shirai, Yasuhiro Torii

**Affiliations:** 1 Department of Comprehensive Dentistry, Graduate School of Medicine, Dentistry and Pharmaceutical Sciences, Okayama University, Okayama, Japan; 2 Center for Education in Medicine and Health Sciences (Dental Education), Graduate School of Medicine, Dentistry and Pharmaceutical Sciences, Okayama University, Okayama, Japan; 3 Comprehensive Dental Clinic, Okayama University Hospital, Okayama, Japan; University of Minho, PORTUGAL

## Abstract

**Objectives:**

We aimed to clarify the communication behaviors between trainee dentists and simulated patients (SPs), to examine how the level of trainee dentists’ self-reported empathy influences assessment by SPs in medical interviews.

**Materials and methods:**

The study involved 100 trainee dentists at Okayama University Hospital and eight SPs. The trainee dentists conducted initial interviews with the SPs after completing the Japanese version of the Jefferson Scale of Empathy (JSE). All interviews were recorded and analyzed using the Roter Interaction Analysis System (RIAS). The SPs assessed the trainees’ communication immediately after each interview. The trainee dentists were classified into two groups (more positive and less positive) according to SP assessment scores.

**Results:**

Compared with less-positive trainees, the more-positive trainees scored higher in the RIAS category of emotional expression and lower in the medical data gathering category. There was no difference in dental data gathering between the two groups. SP ratings for more-positive trainees were higher for use of positive talk and emotional expression and lower for giving medical information and dental information. Trainees with more positive ratings from SPs had significantly higher JSE total scores.

**Conclusion:**

The results of this study suggest that responding to the emotions of patients is an important behavior in dentist-patient communication, according to SPs’ positive assessment in medical interviews. Further, SPs’ assessment of trainees’ communication was related to trainees’ self-reported empathy, which indicates that an empathic attitude among dentists is a significant determinant of patient satisfaction.

## Introduction

Effective communication is a critical determinant of delivering better care to patients. Communication is the process of sharing information between the sender and receiver of a message. Communication is a transactional process and can comprise content and relationship dimensions [1]. Extensive medical literature has suggested that a good communication between a physician and patient has positive effects on patient outcomes, such as increasing patient satisfaction [2,3], reducing anxiety/distress [4], and increasing adherence to treatment [5]. In interviewing patients, it has been reported that specific physician communication behaviors, such as asking about psychosocial issues, socioemotional behaviors, problem defining, and emotion-handling skills are positively related to patient outcome [2–4].

Empathy is an efficient channel of communication with patients and is also considered to be an important determinant of clinical outcomes. Empathy is the ability to put oneself in another’s place, to understand the feelings and problems of another person. It is a complex concept composed of cognitive, emotional, and behavioral elements; however, there is little consensus on the definition of empathy. Hojat defines empathy in the context of patient care, as follows: *“Empathy is a predominantly cognitive (rather than an emotional) attribute that involves an understanding (rather than feeling) of experiences*, *concerns and perspectives of the patient*, *combined with a capacity to communicate this understanding*.*”* [6]. Hojat developed the Jefferson Scale of Empathy (JSE) to measure empathy among physicians and other health providers, focusing on the cognitive dimension. We used the JSE to measure empathy, as we were concerned with the cognitive dimension of empathy. Previous studies have clarified that patients with diabetes who have physicians with high cognitive empathy maintain good metabolic control [7], and patients with HIV whose clinicians have high levels of cognitive empathy demonstrate higher medication self-efficacy [8]. Another study reported that higher physician self-reported empathy is associated with patient satisfaction [9].

The dental context is similar to that of medical relationships. Negative attitudes of dentists in their communication with patients is a significant predictive factor of dental fear [10]. Bernson et al. [11] analyzed interviews with patients who experienced dental-related fear and concluded that verbal and non-verbal communication reflecting empathy was among the main attributes that made dental care accessible to them. In a survey, Imanaka et al. [12] found that communication with dentists was the most important determinant of patient satisfaction in a dental hospital. Armfield et al. [13] also identified that the interpersonal characteristics of dentists and staff members, such as friendliness and respectfulness to patients, were the most common influencers of patient satisfaction. However, contradictory results have been found regarding the relationship between dentists’ communication and patient satisfaction. Sondell et al. [14] reported that dentists’ verbal communication was associated with patient satisfaction only immediately after a specific visit; however, in their earlier study [15], those authors found no association between dentists’ verbal communication and patient satisfaction. These two studies, however, did not investigate which specific dimensions of dentist-patient communication impacted patient satisfaction; these questions need to be further examined.

Although empathic communication in dentistry is assumed to be associated with patient outcomes, anecdotal evidence suggests that collecting insufficient information about a patient’s problem can be one of the antecedents to patient dissatisfaction with their interview. Gathering relevant information and empathic communication are two important aspects in successful medical interviewing. Given this premise, the relationship dimension in communication, especially empathic communication, as well as the content dimension, especially related to information gathering, both influence patient satisfaction with medical interviews.

To the best of our knowledge, no studies have explored the relationship between dentists’ cognitive empathy and patient outcome, except for one study reporting that the overall satisfaction of simulated patients (SPs) with the interview was correlated with dental students’ emotional intelligence [16]. Owing to very few available studies, it remains unclear whether dentists’ self-reported empathy is correlated with patient outcome.

Thus, the aims of this study were 1) to clarify the communication behaviors of trainee dentists, as well as their SPs; and 2) to examine how the level of the trainee dentists’ self-reported empathy influences SPs’ assessment of trainee communication during initial medical interviews.

## Materials and methods

### Participants

We included a total of 100 trainee dentists (47 males and 53 females) enrolled in a 1-year postgraduate clinical training course at Okayama University Hospital in 2015 and 2016 (52 trainees in 2015 and 48 in 2016), and eight SPs from the Okayama Working Group for Simulated Patients (one male and seven females) in this study. Five SPs took part in this study in 2015 and six in 2016, and three SPs participated in both years. Dental education in Japan consists of a 6-year undergraduate program. After acquiring a license, a 1-year postgraduate clinical training program is compulsory.

All trainees provided their written informed consent after receiving an explanation of this study. Their participation was voluntary and did not influence their evaluation in the program. The Ethics Committee of Okayama University Graduate School of Medicine, Dentistry and Pharmaceutical Sciences approved this study (No. 2219).

### Data collection procedure

The trainee dentists conducted initial interviews with the SPs 3 months after the start of clinical training. Each SP carried out the same scenario in the interview. Each SP’s setting was a middle-aged individual with no prior medical history of note. SPs primarily presented concerns about the potential severity of persistent stomatitis on the tongue. Details of the SP scenario are given in S1 Appendix. As SPs were simulated, their responses were contextualized and varied according to the flow of the interviews, which were not standardized. Thus, SPs revealed their concerns whenever they wished to do so. Trainee dentists completed the Japanese version of the JSE for health professionals (HP-Version) immediately before the interviews, which were videotaped and had no time constraints. Immediately after each interview, SPs assessed the trainees’ communication using an assessment sheet. The medical interviews were implemented once yearly in 2015 and 2016. Explanations were given to trainees and SPs in the same manner in both years.

The trainee dentists were classified into two groups, those who were more positive and those who were less positive, based on the median SP assessment score (11.0) of trainees’ communication.

### Measures

#### Assessment sheet: SP assessment of trainee dentists’ communication

The assessment sheet (Table 1) consists of five items measured on a 4-point scale (0 = disagree, 1 = somewhat disagree, 2 = somewhat agree, 3 = agree). Total possible scores range from 0 to 15, where a higher score indicates more positive assessment. This assessment sheet was developed with reference to the American Board of Internal Medicine’s Patient Assessment survey questionnaire, which consists of 10 items [17]. We chose those items that match an initial interview and changed the wording to make it easier for Japanese SPs to understand. Cronbach’s alpha was 0.88, which showed good internal consistency.

**Table 1:** SP assessment sheet.

	How was the dental trainee’s performance at: (circle one number for each item)	Agree	Somewhat agree	Somewhat disagree	Disagree
1	Listening carefully while you are talking?	3	2	1	0
2	Understanding your worry and uneasiness?	3	2	1	0
3	Speaking using appropriate words and speed and plain language?	3	2	1	0
4	Treating you like you are on the same level; never “talking down” to you or treating you like a child?	3	2	1	0
5	Overall, do you want to see this dental trainee?	3	2	1	0

#### The Roter Interaction Analysis System (RIAS): Communication characteristics of trainee dentists during medical interviews with SPs

We analyzed videotaped medical interviews using the RIAS, which is a valid and reliable instrument developed to analyze physician–patient interactions during consultations and is currently one of the most widely used systems of its kind in Western countries [18]. The applicability of the RIAS has also been examined in the Japanese population [19].

In the RIAS, the dialogue between the medical professional and patient is divided into “utterances,” defined as the minimum unit comprising one thought or one piece of information. RIAS categories are composed of two main dimension behaviors, one is task-focused behavior and the other is socioemotional behavior. The former is used to find and resolve problems and includes asking questions and giving information. The latter behavior involves a socioemotional dimension such as building the relationship, engaging with empathic expression, and facilitating conversation [20].

In the Japanese version of the RIAS, each utterance is classified into only one of 42 mutually exclusive code categories. In this study, we added six new categories in the task-focused behaviors, to distinguish dental conversations from other medical conversations; we then concentrated all categories into 14 larger clusters based on similarity of content (Table 2).

**Table 2:** RIAS categories in this study.

Cluster	Categories
Relationship building	Personal remarks, Social conversation, Remediation, Partnership statements, Self-disclosure statement
Positive talk	Laughing, Telling jokes, Showing approval-direct, Giving compliments-general, Showing agreement or understanding, Back-channel responses
Negative talk	Showing disapproval-direct, Criticizing-general
Emotional expression	Empathizing statements, Legitimizing statements, Showing concern or worry, Reassurance, Encouragement or showing optimism, Asking for reassurance
Facilitative behaviors	Giving orientation, Instructing, Paraphrasing/checking for understanding, Asking for understanding, Requesting repetition, Asking for opinions, Asking for permission, Transition words, Requesting services or medication
Counseling/direction	Counseling or direction about any topic
Medical data gathering	Open or Cclosed questions regarding medical conditions or therapeutic regimen
Psychosocial data gathering	Open or closed questions regarding psychosocial or lifestyle issues
Dental data gathering	Open or closed questions regarding current dental history ^a^ or past dental history ^a^
Data gathering about other issues	Open or closed questions about other issues
Medical information giving	Information giving about medical conditions or therapeutic regimen
Psychosocial information giving	Information giving about psychosocial or lifestyle issues
Dental information giving	Information giving about current dental history^a^ or past dental history^a^
Information giving about other issues	Information giving about other issues

^a^ New category.

RIAS coding is done directly from audio or videotapes rather than transcripts. Therefore, utterances can be categorized based on voice tone and phrasing cues, not only literal meaning.

The main coder (S.W.) analyzed all videotapes according to the RIAS manual [18], and 20% of all videotapes (20 tapes, randomly selected) were independently double coded by the second coder (T.Y.), to assess inter-coder reliability. Both coders completed the RIAS coding training offered by RIAS Japan. We calculated inter-class correlation coefficients between results of the two coders for all categories, with a mean frequency greater than two per medical interview. The average correlation was 0.69 (0.25–0.99) for trainee dentists and 0.74 (0.64–0.82) for SPs, which indicated moderate reliability of the coding [21].

We calculated the percentage rates of trainees’ and SPs’ utterances for each category. The overall number of trainee and SP utterances was the denominator, and the number of utterances in each category was the numerator, similar to the calculation methods in other studies [22–26]. The percentage rate was used instead of the absolute number of utterances, to control for interview length.

#### The Jefferson Scale of Empathy (HP-Version): Trainees’ self-evaluation of empathy

The JSE (HP-Version) was developed to measure empathy specifically among physicians and health professionals [27]. Evidence in support of the reliability and validity of the JSE has been previously reported [6]. The JSE has three underlying factors, “perspective-taking”, “compassionate care”, and “standing in the patient’s shoes”, which confirm the latent variable structure [27]. The reliability and validity of the Japanese version of the JSE has been confirmed [28]. Cronbach’s alpha was 0.83 for this population, which showed good internal consistency.

The JSE includes 20 items answered on a 7-point Likert-type scale (1 = strongly disagree, 7 = strongly agree) with a total score range of 20–140. Half of the items are reverse scored. A higher score shows a more empathic orientation toward patient care (Table 3).

**Table 3:** Jefferson scale of empathy (HP-Version).

1	My understanding of how my patients and their families feel is an irrelevant factor in medical treatment.
2	My patients feel better when their physicians understand their feelings.
3	It is difficult for me to view things from my patients’ perspectives.
4	I consider understanding my patients’ body language as important as verbal communication in physician–patient relationships.
5	I have a good sense of humor, which I think contributes to a better clinical outcome.
6	Because people are different, it is almost impossible for me to see things from my patients’ perspectives.
7	I try not to attention to my patients’ emotions in interviewing and history taking.
8	Attentiveness to my patients’ personal experiences is irrelevant to treatment effectiveness.
9	I try to imagine myself in my patients’ shoes when providing care to them.
10	My understanding of my patients’ feelings gives them a sense of validation that is therapeutic in its own right.
11	Patients’ illnesses can be cured only by medical treatment; therefore, affectional ties to my patients cannot have a significant place in this endeavor.
12	I consider asking patients about what is happening in their lives as an unimportant factor in understanding their physical complaints.
13	I try to understand what is going on in my patients’ minds by paying attention to their nonverbal cues and body language.
14	I believe that emotion has no place in the treatment of medical illness.
15	Empathy is a therapeutic skill without which my success as a physician would be limited.
16	An important component of the relationship with my patient is my understanding of the emotional status of the patients and their families.
17	I try to think like my patients in order to render better care.
18	I do not allow myself to be touched by intense emotional relationships between my patients and their family members.
19	I do not enjoy reading nonmedical literature and the arts.
20	I believe that empathy is an important therapeutic factor in medical treatment

### Statistical analyses

The mean percentages of trainees’ and SPs’ utterances for each category, the total JSE score, and the length of the medical interview were compared between trainee dentists who had more-positive or less-positive assessments by SPs. Because the percentage rates of utterances were not expected to be normally distributed, the Mann-Whitney test was used. Unpaired *t*-tests were used for total JSE scores and the length of the medical interview because the data were normally distributed.

All analyses were performed using IBM SPSS version 23 (IBM, Tokyo, Japan), and the significance level was set to 0.05.

## Results

### SP assessment of trainee dentists’ communication

**Fig 1:**
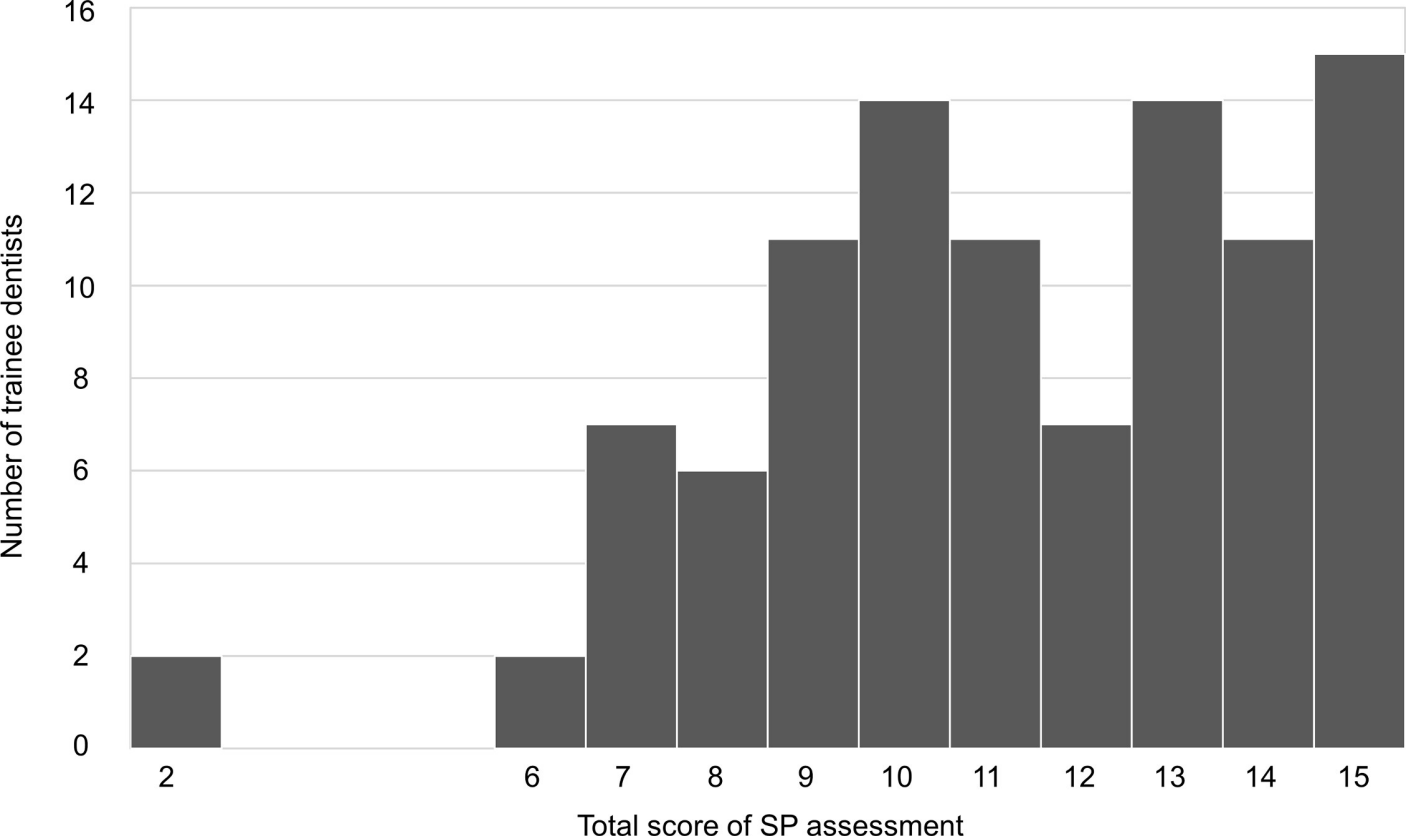
Distribution of total scores for SP assessment.

The distribution of the total scores for SP assessment is shown in Fig 1. The mean total score was 11.2 and the median score was 11.0 (SD, 2.9; range, 2–15). We used the median score as a base value and included 11.0 in the low group, to yield a participant number in each group that was as equal as possible. Trainee dentists with SP assessment scores ≥12 were classified as the more-positive group (n = 47), and those with scores <12 were classified as the less-positive group (n = 53).

### RIAS

#### Percentage rates of the trainees’ and SPs’ utterances

The mean percentage rates and mean frequencies of trainees’ and SPs’ utterances for the clusters are given in Tables 4 and 5, respectively. We expressed the cluster names in brackets in this study. Compared with the trainee dentists whose SP assessment was less positive, those with more-positive assessments had greater [Emotional expression], especially empathic and legitimizing statements. However, this group had a lower proportion for [Medical data gathering]. There was no difference in [Dental data gathering] between the two groups.

**Table 4:** Mean proportions and mean frequencies of clusters in RIAS categories for trainee dentists with more-positive and less-positive SP assessments.

	More positive n = 47			Less positive n = 53			P-value
	Mean%	(Mean N)	SD	Mean%	(Mean N)	SD	
Total utterances	54.95%	(115.7)	4.32%	55.63%	(90.5)	3.74%	0.419
Relationship building	3.75%	(4.1)	1.35%	4.37%	(3.8)	1.63%	0.076
Positive talk	43.15%	(50.1)	7.47%	41.17%	(38.0)	9.01%	0.373
Negative talk	0.00%	(0.0)	0.00%	0.02%	(0.0)	0.15%	0.346
Emotional expression	2.13%	(2.6)	1.90%	0.93%	(0.9)	1.29%	0.000**
Facilitative behaviors	26.78%	(30.8)	5.07%	26.57%	(23.9)	7.03%	0.841
Counseling/direction	0.00%	(0.0)	0.00%	0.00%	(0.0)	0.00%	1.000
Medical data gathering	7.30%	(8.4)	2.72%	8.77%	(7.8)	4.16%	0.026*
Psychosocial data gathering	1.45%	(1.8)	1.33%	1.58%	(1.4)	1.36%	0.584
Dental data gathering	15.08%	(17.5)	4.48%	16.42%	(14.5)	4.32%	0.107
Data gathering tfor other issues	0.00%	(0.0)	0.00%	0.00%	(0.0)	0.00%	1.000
Medical information giving	0.15%	(0.2)	0.40%	0.08%	(0.1)	0.36%	0.150
Psychosocial information giving	0.02%	(0.0)	0.12%	0.01%	(0.0)	0.10%	0.921
Dental information giving	0.20%	(0.2)	0.69%	0.07%	(0.1)	0.26%	0.554
Information giving for other issues	0.00%	(0.0)	0.00%	0.00%	(0.0)	0.00%	1.000

*P-value <0.05

**P-value <0.01.

N: number of the utterances.

**Table 5:** Mean proportion and mean frequency of clusters in RIAS categories for SPs who assessed trainee dentists more positively and less positively.

	More positive n = 47			Less positive n = 53			P-value
	Mean %	(Mean N)	SD	Mean%	(Mean N)	SD	
Total utterances	45.05%	(95.9)	4.32%	44.37%	(72.0)	3.74%	0.419
Relationship building	2.28%	(2.1)	1.24%	2.46%	(1.7)	1.55%	0.468
Positive talk	44.31%	(43.1)	8.34%	39.51%	(29.0)	7.25%	0.007**
Negative talk	0.61%	(0.7)	0.87%	0.63%	(0.5)	1.08%	0.697
Emotional expression	1.22%	(1.2)	1.05%	0.44%	(0.3)	0.86%	0.000**
Facilitative behaviors	1.45%	(1.4)	1.68%	1.65%	(1.1)	1.72%	0.482
Counseling/direction ^a^	-	-	-	-	-	-	-
Medical data gathering	0.09%	(0.1)	0.30%	0.04%	(0.0)	0.26%	0.141
Psychosocial data gathering	0.03%	(0.0)	0.20%	0.00%	(0.0)	0.00%	0.288
Dental data gathering	0.11%	(0.1)	0.36%	0.07%	(0.1)	0.30%	0.579
Data gathering for other issues	0.00%	(0.0)	0.00%	0.00%	(0.0)	0.00%	1.000
Medical information giving	12.00%	(11.2)	4.02%	13.72%	(9.7)	5.17%	0.031*
Psychosocial information giving	7.34%	(7.2)	3.43%	6.65%	(4.8)	4.03%	0.203
Dental information giving	30.51%	(28.8)	6.42%	34.82%	(24.7)	6.81%	0.001**
Information giving for other issues	0.04%	(0.0)	0.21%	0.02%	(0.0)	0.16%	0.491

*P-value <0.05.

**P-value <0.01.

^a^ Category for dentists only.

N: number of utterances.

SPs gave higher ratings for [Positive talk], especially back-channel response, and [Emotional expression], which includes concerns of the patient, in the interviews with more-positive trainees. However, the rates of [Medical information giving] and [Dental information giving] were lower in the positive group.

### JSE

**Fig 2:**
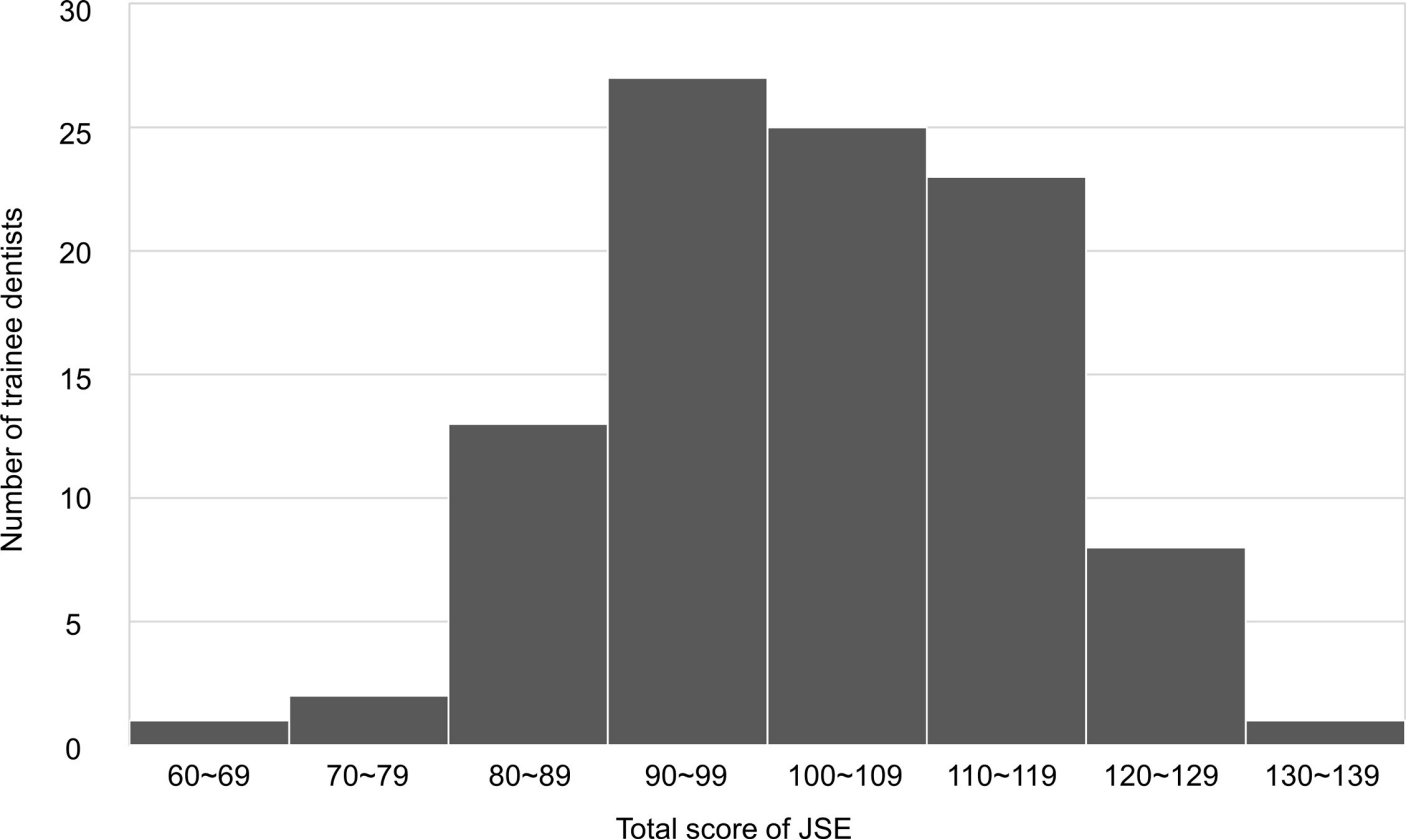
Distribution of total scores for the JSE.

The distribution of total scores for the JSE is displayed in Fig 2. The mean total JSE score was 102.00 (SD, 12.5; range, 64–132). There was a significant difference in JSE total scores between the more-positive and less-positive groups (104.8 vs. 99.5). There was no significant difference between female and male trainee dentists in this study (102.7 vs. 101.4).

### Length of the medical interview

The mean interview length was 8 min 34 s (SD,2 min 30 s, range 3 min 43 s to 17 min 56 s). The mean length of the interviews with more-positive trainee dentists was significantly longer than that with less-positive ones (9 min 34s vs. 7 min 40 s).

The results indicated that SPs regarded trainees who conducted longer interviews more favorably.

## Discussion

Our finding of a positive relationship between trainee dentists’ as well as SP’s empathic communication and SPs’ assessment suggests that recognizing patients’ emotions and expressing acceptance of these emotions using verbal or nonverbal communication leads patients to open up and to be more active conversationally. Such empathy was received positively by SPs and led to higher assessments of trainees. This is a reciprocal interaction. Some prior studies have showing findings similar to this result. Roter et al. [29] investigated the effects of communication skills training and found that trained doctors used more facilitation and tended to engage more in emotional talk. Their patients also tended to use more positive statements and report higher satisfaction than the patients of untrained doctors. Dulmen et al. [30] found that more anxious patients preferred empathic doctors and that empathic responses were rated as appropriate responses by doctors. The fact that the SPs in our study expressed concerns might reflect this finding.

In the dental context, our findings were partly consistent with studies among prosthodontic patients reporting that dentist communication was associated with patient satisfaction only immediately after a specific visit but not with overall patient satisfaction in the longer term [14]. This may imply that observable dentist–patient communication may not be directly related to treatment outcomes, which reflects many characteristics of dentistry. A consultation in dentistry is nearly always accompanied by a subsequent invasive procedure, and manual skills also affect treatment success, such as in prosthodontics, endodontics, and oral surgery. Patient satisfaction regarding clinical treatment quality has received much attention and is often assessed in the dental literature [31]. The SPs in our study assessed dentists’ communication only during their interviews, which may affect our finding that dentists with empathic communication received higher assessments from SPs.

Our study results indicated that trainees with positive assessments had a lower or equal rate of utterances in [Medical data gathering] and [Dental data gathering]; however, in terms of the number of utterances, more-positive trainees and their SPs had a greater number of utterances regarding medical and dental data exchange compared with those in the less-positive group, even though this was not statistically tested. Thus, this result does not mean that more-positive trainees and their SPs gathered or gave less biomedical information but it does suggests that more utterances were allotted to [Emotional expression], such as responding and showing emotions, than to gathering and giving information, in comparison with those in the less-positive group. Gathering relevant information is indispensable for accurate diagnosis, and responding to patients’ emotions is an important characteristic for patient satisfaction. Both aspects are needed for successful medical interviews; therefore, the interviews of trainee dentists who received more-positive assessments from SPs took more time than those of less-positive trainees.

Another notable finding was that the trainees’ self-reported empathy was positively related with the ratings from SPs. An earlier study agreed with our findings [32]; however, another study showed contradictory results [33]. These two studies, however, measured SPs’ perception of students’ or residents’ empathy, which is not exactly the same as what we measured. In our study, SPs evaluated trainees’ communication, which may suggest that trainees demonstrated an empathic attitude in their communication with SPs, and SPs perceived that empathy. We found that trainees who received more-positive assessments used more empathic communication, which may indicate that cognitive measures of empathy may reflect behavioral measures. One study reported the relation between self-reported empathy and communication behavior in emotional responsiveness [34]; however, another study reported contradictory findings [35]. We did not examine the relationship between trainees’ self-reported empathy and their communication behavior; further studies are needed to confirm the positive relationship between these two measures.

Considering our findings, patient satisfaction can be improved by increasing the dentist’s empathy. As the existing literature has demonstrated the effectiveness of enhancing clinicians’ empathy [36], it seems that empathy can be taught and learned. Therefore, it is worth emphasizing development of an empathic attitude toward patients in the dental curriculum.

This study had some limitations. First, because our study involved only one institution and had a small sample size, we cannot eliminate the impact of sex concordance between dentists and SPs. Second, we only analyzed communication during the initial interview encounter and not during the entire clinical interaction. Thus, the results of SP assessment in this study only partly reflect patients’ satisfaction. Third, we included non-verbal behaviors with utterances but did not include non-verbal behaviors without utterances in our analysis, which may limit our results. Last, we only compared the number of communication behaviors and did not take into account the order in which communication behaviors were seen. These limitations may limit the ability to generalize these findings to a wider population. Verification of these findings in future studies is warranted.

## Conclusion

In this study, we provided evidence that responding to patients’ emotions is an important behavior in dentist-patient communication, for positive assessment by patients. For this reason, the conversation during medical interviews in dental settings should not be restricted to biomedical topics but should also include responding to the patient’s emotional statements. Additionally, we found that SPs’ assessment of trainees’ communication was related to trainees’ self-reported cognitive empathy. These findings add to the body of literature indicating that promoting an empathic attitude is an important aspect in the dental education curriculum.

## Supporting information

S1 Appendix SP senario. (DOCX)

## References

[1] Northourse LL, Northouse PG. Health communication: strategies for health professionals. Stamford: Appleton & Lange; 1998.

[2] Bertakis KD, Roter D, Putnam SM. The relationship of physician medical interview style to patient satisfaction. J Fam Pract. 1991;32: 175–181. 1990046

[3] Ong LM, Visser MR, Lammes FB, de Haes JC. Doctor-patient communication and cancer patients’ quality of life and satisfaction. Patient Educ Couns. 2000;41: 145–156. 12024540 10.1016/s0738-3991(99)00108-1

[4] Roter DL, Hall JA, Kern DE, Barker LR, Cole KA, Roca RP. Improving physicians’ interviewing skills and reducing patients’ emotional distress. A randomized clinical trial. Arch Gen Intern Med. 1995;155: 1877–1884. 10.1001/archinte.1995.00430170071009 7677554

[5] Wachira J, Middlestadt S, Reece M, Peng CY, Braitstein P. Physician communication behaviors from the perspective of adult HIV patients in Kenya. Int J Qual Health Care. 2014;26: 190–197. 10.1093/intqhc/mzu004 24519123

[6] Hojat M. Empathy in patient care: antecedents, development, measurement, and outcomes. Philadelphia: Springer; 2007.

[7] Hojat M, Louis DZ, Markham FW, Wender R, Rabinowitz C, Gonnella JS. Physicians’ empathy and clinical outcomes for diabetic patients. Acad Med. 2011;86: 359–364. 10.1097/ACM.0b013e3182086fe1 21248604

[8] Flickinger TE, Saha S, Roter D, Korthuis PT, Sharp V, Cohn J, et al Clinician empathy is associated with differences in patient-clinician communication behaviors and higher medication self-efficacy in HIV care. Patient Educ Couns. 2016;99: 220–226. 10.1016/j.pec.2015.09.001 26395313 PMC5610904

[9] Wang H, Kline JA, Jackson BE, Laureano-Phillips J, Roinson RD, Cowden CD, et al Association between emergency physician self-reported empathy and patient satisfaction. PLoS One. 2018;13: e0204113 10.1371/journal.pone.0204113 30212564 PMC6136813

[10] Abrahamsson KH, Hakeberg M, Stenman J, Öhrn K. Dental beliefs: evaluation of the Swedish version of the revised Dental Beliefs Survey in different patient groups and in a non-clinical student sample. Eur J Oral Sci. 2006;114: 209–215. 10.1111/j.1600-0722.2006.00358.x 16776770

[11] Bernson JM, Hallberg LR, Elfström ML, Hakeberg M. 'Making dental care possible: a mutual affair': a grounded theory relating to adult patients with dental fear and regular dental treatment. Eur J Oral Sci. 2011;119: 373–380. 10.1111/j.1600-0722.2011.00845.x 21896054

[12] Imanaka M, Nomura Y, Tamaki Y, Akimoto N, Ishikawa C, Takase H, et al Validity and reliability of patient satisfaction questionnaires in a dental school in Japan. Eur J Dent Educ. 2007;11: 29–37. 10.1111/j.1600-0579.2007.00438.x 17227393

[13] Armfield JM, Enkling N, Wolf CA, Ramseier CA. Dental fear and satisfaction with dental services in Switzerland. J Public Health Dent. 2014;74: 57–63. 10.1111/j.1752-7325.2012.00368.x 22970925

[14] Sondell K, Palmqvist S, Söderfeldt B. The dentist’s communicative role in prosthodontic treatment. Int J Prosthodont. 2004;17: 666–671. 15686094

[15] Sondell K, Söderfeldt B. Palmqvist S. Dentist-patient communication and patient satisfaction in prosthetic dentistry. Int J Prosthodont. 2002;15:28–37. 11887596

[16] Hannah A, Lim BT, Ayers KM. Emotional intelligence and clinical interview performance of dental students. J Dent Educ. 2009;73: 1107–1117. 19734253

[17] Abadel FT, Hattab AS. Patients’ assessment of professionalism and communication skills of medical graduates. BMC Med Educ. 2014;14: 28 10.1186/1472-6920-14-28 Available from: https://bmcmededuc.biomedcentral.com/articles/10.1186/1472-6920-14-28 24517316 PMC3923249

[18] Noro I, Abe K, Ishikawa H. The Roter Method of Interaction Process Analysis System (RIAS). 2nd ed Aichi: Sankeisha; 2011. Japanese.

[19] Ishikawa H, Takayama T, Yamazaki Y, Seki Y, Katsumata N. Physician-patient communication and patient satisfaction in Japanese cancer consultations. Soc Sci Med. 2002;55: 301–311. 10.1016/S0277-9536(01)00173-3 12144143

[20] Roter D, Larson S. The Roter interaction analysis system (RIAS): utility and flexibility for analysis of medical interactions. Patient Educ Couns. 2002;46: 243–251. 11932123 10.1016/s0738-3991(02)00012-5

[21] Koo TK, Li MY. A guideline of selecting and reporting intraclass correlation coefficients for reliability research. J Chiropr Med. 2016;15: 155–163. 10.1016/j.jcm.2016.02.012 27330520 PMC4913118

[22] Ishikawa H, Takayama T, Yamazaki Y, Seki Y, Katsumata N, Aoki Y. The interaction between physician and patient communication behaviors in Japanese cancer consultations and the influence of personal and consultation characteristics. Patient Educ Couns. 2002;46: 277–285. 10.1016/S0738-3991(01)00164-1 11932127

[23] Hanya M, Kanno Y, Akasaki J, Abe K, Fujisaki K, Kamei H. Effects of communication skill training (CST) based on SPIKES for insurance-covered pharmacy pharmacists to interact with simulated cancer patients. J Pharm Health Care Sci. 2017;3: 11 10.1186/s40780-017-0080-0 28405409 PMC5385086

[24] Sondell K, Söderfeldt B, Palmqvist S. A method for communication analysis in prosthodontics. Acta Odontol Scand. 1998;56: 48–56. 10.1080/000163598423063 9537735

[25] Ford S, Fallowfield L, Lewis S. Doctor-patient interactions in oncology. Soc Sci Med. 1996;42:1511–1519. 10.1016/0277-9536(95)00265-0 8771634

[26] Hull FM, Hull FS. Time and the general practitioner: the patient’s view. J R Coll Gen Pract. 1984;34: 71–75. 6471020 PMC1959600

[27] Hojat M, Gonnella JS, Nasca TJ, Mangione S, Vergare M, Magee M. Physician empathy: definition, components, measurement, and relationship to gender and specialty. Am J Psychiatry. 2002;159: 1563–1569. 10.1176/appi.ajp.159.9.1563 12202278

[28] Kataoka HU, Koide N, Hojat M, Gonnella JS. Measurement and correlates of empathy among female Japanese physicians. BMC Med Educ. 2012;12: 48 10.1186/1472-6920-12-48 22726449 PMC3493267

[29] Roter D, Rosenbaum J, de Negri B, Renaud D, DiPrete-Brown L, Hernandez O. The effects of a continuing medical education programme in interpersonal communication skills on doctor practice and patient satisfaction in Trinidad and Tobago. Med Educ. 1998;32: 181–189. 10.1046/j.1365-2923.1998.00196.x 9766977

[30] van Dulmen S, van den Brink-Muinen A. Patients’ preferences and experiences in handling emotions: a study on communication sequences in primary care medical visits. Patient Educ Couns. 2004;55: 149–152. 10.1016/S0738-3991(04)00300-3 15540370

[31] Alfadda SA, Al Amri MD, Al-Ohali A, Al-Hakami A, Al-Madhi N. Two-implant-supported mandibular overdentures: do clinical denture quality and inter-implant distance affect patient satisfaction? Int J Prosthodont. 2017;30: 519–525. 10.11607/ijp.5295 29084295

[32] Berg K, Majdan JF, Berg D, Veloski J, Hojat M. A comparison of medical students’ self-reported empathy with simulated patients’ assessments of the students’ empathy. Med Teach. 2011;33: 388–391. 10.3109/0142159X.2010.530319 21517687

[33] Grosseman S, Novack DH, Duke P, Mennin S, Rosenzweig S, Davis TJ, et al Residents’ and standardized patients’ perspectives on empathy: issues of agreement. Patient Educ Couns. 2014;96: 22–28. 10.1016/j.pec.2014.04.007 24793008

[34] LaNoue MD, Roter DL. Exploring patient-centeredness: the relationship between self-reported empathy and patient-centered communication in medical trainees. Patient Educ Couns. 2018;101: 1143–1146. 10.1016/j.pec.2018.01.016 29395476

[35] Chen DC, Pahilan ME, Orlander JD. Comparing a self-administered measure of empathy with observed behavior among medical students. J Gen Intern Med. 2010;25: 200–202. 10.1007/s11606-009-1193-4 20013070 PMC2839329

[36] Batt-Rawden SA, Chisolm MS, Anton B, Flickinger TE. Teaching empathy to medical students: an updated, systematic review. Acad Med. 2013;88: 1171–1177. 10.1097/ACM.0b013e318299f3e3 23807099

