# Supplementary material for: Relationship of trainee dentists’ self-reported empathy and communication behaviors with simulated patients’ assessment in medical interviews
Source: PLoS One. 2018 Dec 20;13(12):e0203970. doi: 10.1371/journal.pone.0203970 (PMC6301708; doi:10.1371/journal.pone.0203970)
Supplement: S1 Appendix — (DOCX) [file pone.0203970.s001.docx]

SP scenario (female version)

History of present illness

Mrs. (Mr.) Maehara took out her crown (the second tooth from the back on the bottom left) about 2 years ago. Sicne then she has suffered form stomatitis on the left side of her tongue. It stung when she ate something sour, or hot so that she could not enjoy eating. The pain had lessened sometimes. She had to take care of her mother, so that she couldn’t have time to visit a dentist and she left it as it was.

She finally visited a dentist, started the treatment last fall and got a bridge installed the end of the year. The dentist told her that after the crown came off, the remaing teeth rubbed on her tongue, and that caused the stomatitis. The dentist prescried s topical cream, and she used it. Thanks for that the level of pain decreased, but the stomatitis still remains. Also, it turned white in color and hardend.

She recently saw a TV show about stomatitis. They were advising one to go get checked if the stomatitis persistently don’t heal because it could be cancer. She became worried and decided to visit an outpatient clinic at Okayama University Hospital because it is a major hospital and have her condition very thoroughly.

Patient’s perspective toward the present problems

She believes that the stomatitis was caused by the jagged edge of her tooth. However, the information that was talking on TV made her uneasy. She has no other problems in her mouth.

Past dental and medical history

Mrs.(Mr.) Maehara had a crown (the second tooth from the back on the bottom left) at a nearby dentist about 10 years ago. She has never undergone dental treatment for another tooth. She does not visit a dentist regularly. She has never had a bad reaction during dental treatment.

She is healthy. She has no allergies. She has never been hospitalized.

Oral health behavior

She brushes her teeth twice a day.

Social history

She has no chmoved to Okayama from Kurashiki 7 years ago. She married a colleague from the same workplace, she became a housewife after marriage. She has no children. She smokes and drinks a little.
